# Dynamic and Functional Approach to Human Memory in the Brain: A Clinical Neuropsychological Perspective

**DOI:** 10.3389/fpsyg.2017.00688

**Published:** 2017-05-04

**Authors:** Yannick Gounden, Mathieu Hainselin, Fabien Cerrotti, Véronique Quaglino

**Affiliations:** CRP-CPO, EA 7273, Université de Picardie Jules VerneAmiens, France

**Keywords:** memory, neuropsychology, assessment, model, structural

## Abstract

The way how cognition is conceived and represented in brain functioning will directly impact clinical investigations of people with cognitive difficulties. This is particularly evident in the field of clinical neuropsychology where methodologies and tools are justified on a fundamental level by the theoretical foundations adopted. The present article outlined how the dominant influences of structural and anatomo-clinical theories of memory have led to a particular conception of clinical investigations. We propose to reconsider these dominant methods in favor of a more dynamic and functional representation of memory that would be clinically more appropriate. More precisely, we argued that relying exclusively on a particular memory conception (i.e., structural) may not be sufficient considering the range of real-life variables affecting a patient’s memory. By extracting clinically meaningful information in more functional and dynamic memory conceptions, we also aim at underlining the potentials advantages of such theories in facilitating personalized assessments and follow up of patients in clinical neuropsychology. We suggest that a dynamic, functional, and integrative conception of memory would be more coherent with the trend in clinical neuropsychology to promote a more collaborative interaction between the clinician and the patient. Finally, considering the absence of empirical studies on the possible benefits of implementing such recent memory concepts in clinical practice, we encourage researchers and clinicians to test in the field of clinical neuropsychology, the usefulness and explanatory power of more dynamic and functional representation of memory in order to objectively demonstrate its validity outside the research loop.

## Memory Representations

Neuropsychology is a discipline at the interface of neurosciences, cognitive and clinical psychology and it studies the mind–brain relationship. Different conceptions have contributed in understanding how memory works by identifying the processes and their underlying anatomical structures. While cognitive psychology addresses cognitive processes by decomposing them into modular subsystems (Fodor, 1986), research in neuroscience has identified dynamic brain changes as functional supports of memory traces (Nader, 2003). The question of whether cognition (and particularly memory) is divided into specific systems corresponding to dedicated brain structures or if it is represented by different and multiple processes in a holistic and dynamic organization of brain function has frequently been subject to discussion (Shallice, 1988). In the field of clinical neuropsychology, memory processes are most likely conceived as the result of coordinated activities of different subsystems. This conception of memory into multiple systems that differ structurally and functionally has been encouraged by the observation of double dissociation of cognitive disorders. For instance, in such conception one may have a selective deficit affecting only a particular memory subsystem, while another person may show the reverse pattern. Observation of such double dissociation was seen as an argument supporting the independence assumption among the various memory subsystems (Tulving, 1985). Previous conceptions of memory, like those proposed by functionalists (Craik and Lockhart, 1972; Nairne and Pandeirada, 2010), still remain in the research loop.

The structural model of Tulving (Tulving, 1972, 1995, 2002) currently known as the SPI model (Serial-Parallel-Independent; for serial encoding, parallel storage, and independent retrieval; Tulving, 1995) is probably the most influential memory model in both research and clinical domains. It provides a description of memory as multiple systems that differ structurally and functionally. Episodic memory allows to travel from past to future and to remember the event (what) and its context (Tulving, 2002). Semantic memory contains general facts about the world, and the Perceptual Representation System (PRS) allows the priming effects. Originally, these distinctions were based on the different types of information processed by the different systems (for example: information with unique spatial-temporal contexts for episodic memory and facts and concepts for semantic memory). However, later Tulving (2002) emphasized on the level of consciousness (the experience of remembering) associated to each memory system: autonoetic consciousness for episodic memory, noetic consciousness for semantic memory and anoetic consciousness for procedural memory. Moreover, initially the various memory systems were considered as relatively independent but in the light of later findings, Tulving (2005) suggests the existences of possible interactions between the memory systems (for instance, semantic memory and episodic memory are seen as highly interactive and complementary systems).

The structural division of human memory has been challenged by current knowledge, which defends a conception of a unique structured memory as parallel mental processes widely distributed over the brain. Besides refuting the existence of multiple systems, they also emphasize on the dynamic, constructive and adaptive nature of memory. As a reminder, according to classical structuraliste model of memory, during consolidation (the stabilization process of information in memory), the newly learnt information is fragile and labile with the possibility of being disrupted. However, once consolidation is done, memory for the information is posited to be more or less permanent, more resistant to interference and more likely to be recollected later on (Lane et al., 2015). Inversely, in a more dynamic conception of memory, consolidation does not give rise to memory representations that are immutable records of an event. Instead, the passing of time and repeated recollections are posited to render the previously consolidated information again labile and subject to continuous modifications and reconsolidation.

For instance, Nadel and Moscovitch (1997) proposed a memory theory (Multi Trace Theory) that considers the dynamic relation between retrieval and reconsolidation. Unlike the classical structural memory model, the Multi Trace Theory (MTT) framework, argues that the hippocampus remains critically involved in old episodic memories, and that over time its participation even increases. Similarly, memory trace (engram) would extend with each activation and reactivation of memory. Thus, repeated co-activation between different neocortical modules would gradually link these separate modules. The creation of traces, linked by common elements, facilitates the extraction of knowledge tied to common experiences. This mechanism appears either by strengthening existing connections or by generating new nodes or neural units. The interactions between brain regions and memory processes are thus transient. Memory traces, encounter thereby various transformations over time: the former could be reactivated, new would be built in encoding and integrated into the existing network. Such integration could occur by following consolidation schemes in the neocortex (Tse et al., 2007). The information could ultimately be different from the initial one and engender forgetfulness, interference and other recovery related problems (distortions). The reactivation of memory can thus be strengthened by specific reproduction or expansion of neural traces, but may also lead to instability of the trace to allow reorganization and rearrangement to incorporate new information (Nadel and Hardt, 2011; Nadel et al., 2012; Moscovitch et al., 2016). The conception of memory as a changing organization in the form of traces which reflect the brain’s dynamic reality in the tangle of other cognitive functions is well ascribed in research field. However, such conception has not yet gained its milestones in the dynamic context of clinical investigation of memory.

## Questioning The Typical Clinical Investigation Of Memory

Training of clinical psychologists has greatly been influenced by a scientist-practitioner model (Baker and Benjamin, 2000). Ideally, clinicians are encouraged to translate research findings into clinically meaningful information in order to have a clinical practice in line with up to date findings. However, in reality there is a gap between research and clinical practice (Beutler et al., 1995). This is undoubtedly true when considering the field of memory investigation. The prevailing way of proceeding in clinical practice is to draw links between deficits of a particular memory system and certain brain structures. This may be allotted to the (almost exclusively) structure/deficits training and to the fact that in clinical domain, patients usually undergo structural brain investigations and rarely functional dynamic investigations (i.e., looking for a complex memory brain network). When conducting neuropsychological assessments, clinicians are confronted to many real-life variables. Actually, the dynamic models of memory have limited impact in the practice of clinicians (Nadel et al., 2000; Moscovitch et al., 2005; Barsalou, 2008; Moyal-Sharrock, 2009; Eustache et al., 2016) and to our knowledge there is no empirical evidence on the benefits of implementing such conception in clinical practice. In our point of view, the problem is not that practitioners pay little heed to research findings, but in part due to ongoing debates among researchers defending a particular memory conception (Moyal-Sharrock, 2009; Nairne and Pandeirada, 2010). Debating is indeed important but may not be directly relevant to real-world practice where the primary aim of clinicians is to improve care.

Structuralist models are interesting for levels of description and are meaningful for practitioners in various ways, for example, with the SPI model, the neuropsychological differentiation between forms of dementia can be seen in the division of memory systems, with selective preservation and loss of memory skills (De Deyn and D’Hooge, 2003). However, these models lose their descriptive power when it comes to neurofunctional mapping. To illustrate our statement, let’s take as example the conception of encoding and retrieval functions. The SPI model defines retrieval from its different systems as independent. In other words, the encoding of information at a level of representation is not influenced by the retrieval of information at another level and vice versa. On a cerebral point of view, this conception does not seem to be supported. Indeed, many studies currently support a more integrated and highly interactive view of the representation of memory in the brain. The flow of neural information from inferior systems to superior systems of information processing (i.e., perception/encoding) is constantly predicted by a descendant influence on the basis of past experience (i.e., recovery), itself influenced by the quality/quantity of information from the lower systems (Friston, 2005; Gagnepain, 2011). Thus, in adopting a strict structural analysis of memory, clinical neuropsychologists may miss the dynamic nature of memory, thereby neglecting also its adaptive role.

It is flagrant in various fields of memory research like in neuroscience, cognitive psychology and clinical neuropsychology, that the primary focus of researchers is largely on “How” things are remembered, and less likely on “Why” a particular information is more likely to be remembered than another (Nairne and Pandeirada, 2010). Although interest for the “Why” problematic can be seen in a few domains such as memory for distinctive information (Hunt and Worthen, 2006) the structural multiple systems approach prevails.

The structural approach enables us to response to questions such as “What did you see on TV or Where is the Eiffel tower found?” Most of the memory tests used by clinical neuropsychologists also focus on the recalling of information in response to “What or Where” question. For instance, the Free and Cued Selective Reminding Test (Buschke, 1984) which is almost systematically used in memory centers (at least in France), requires the examinee to recall 16 words previously presented. By investigating on the person’s capacity in finding the words (response to a “What” question), the clinician can have information on “How” a particular memory system is efficient or not. In case of memory difficulties in encoding, storing or retrieving past information, the clinician may propose various aids targeting a particular step depending on the nature of the problem (Encoding? Retrieval…?) or a particular memory system (De Deyn and D’Hooge, 2003).

However, as noted by functionalists (Nairne and Pandeirada, 2008, 2010), the past can never be experienced again or at least in exactly the same way. Thus, investigating on “How” an information is memorized without considering “Why,” is to our point of view reductive. When assessing memory, clinicians should also pay attention to “Why” an information is retained or forgotten. Events occurring in our environment do not have the same weight. Some are more distinctive than others. The distinctive nature of an event may also be of different nature (emotional, visual, …) and may vary from an individual to another and even within the same individual at different moments due to various factors (emotional ties, cultural factors, motivation…) (Hunt and Worthen, 2006). In taking this aspect in consideration, clinical neuropsychologists may have a better insight of their patients and propose more personalized care. Moreover, by considering in conjunction the “How” and the “Why,” such an integrative perspective of memory may also contribute in understanding the dynamic nature of “Where” things occur in the brain.

## A Plead for A More Dynamic, Functional and Integrative Approach to Memory in Clinical Practice

The present work outlines why relying exclusively on a particular memory conception (i.e., structural) could pigeonhole clinical neuropsychologists when caring for patients. By extracting clinically meaningful information in various memory conceptions, we also encourage practitioners to adopt a more dynamic and integrative approach which could be more clinically relevant.

Neurobiological models place memory in the brain’s dynamic reality in the tangle of cognitive functions involved in a particular task. The interactions between brain regions and memory processes are posited to be implemented according to behavior and environment, and are thus subject to change (Nadel et al., 2000; Moscovitch et al., 2005, 2016). In this perspective, forgetting is not exclusively due to a deficit of (or in accessing) a memory system in the brain. The phenomenon can also be explained in terms of a trace activation default or mistakes, which can be the consequences of a wide possibility of factors (personal, emotional, social …). However, this conception is almost missing in clinical practice. In assessing memory, the dynamic nature of organization and reorganization of memory (which can be an adaptive response) is too rarely considered. Moreover, the various tools such as the widely used Free and Cued Selective Reminding Test (Buschke, 1984) is not devised to take into consideration the natural dynamic nature of memory in the context of a real memory impairement. “Why does the patient suffering from Alzheimer disease, still remember this particular word, person, task…weeks later?” “Why such reconstrcution or memory distortions?” “Why is his or her memory profil different form the other patient with the same disease?”

Although taking into consideration the number of recalled words at a given test is important, relying only on this indicator is too reductive of “How” memory functions. Indeed, the “How” and the “Why” an information is encoded or retrieved, should both be considered in conjunction if we aim at considering the singularity of our patient. Moreover, while recognizing that certain brain regions are particulary involved in memory, it is also necessary to bear in mind the dynamic nature of “Where” it happens in the brain.

The advantage of adopting a dynamic conception of memory can be seen in other domains of psychology (such; behavioral therapy, cognitive-behavioral therapy, psychodynamic psychotherapy and emotion-focused therapy) where such conception is central in mediating therapeutic change. More precisely, it is argued that the reactivation of old memories to include new emotional experiences via the process of reconsolidation is essential in the process of therapeutic change (Lane et al., 2015). Likewise, in the field of clinical neuropsychology, the emotion-memory interaction is a factor of importance along with other variables such as to name a few: social, cultural, and of course the type of pathology (organic and/or psychological disturbances).

As stated previously, to our knowledge, there is currently no empirical evidence on the benefits of implementing dynamic and integrative memory conceptions in clinical practice. Concerning memory assessments, we believe that such a conception can in addition to quantitative data that is “the how of remembering” (the number of recalled items, number of errors…), also encourage the considerations of valuable qualitative information such as the type of interferences, the nature of distortions, confabulations, motivation, anxiety during testing,…etc. Indeed, a more dynamic conception of memory where interest is also given to the “Why of remembering” can have the advantage of bringing neuropsychologists to have a more personalized interpretation of a memory profile beyond the typical normal-pathological distinction based on test scores. This more refined analysis of memory profile is possible because memory in a dynamic conception is not perceived as faithful records of the past but something that is flexible, contextually bound, and subject to modifications triggered by personal experiences including the neuropsychological assessment context itself. However, for exploiting the potentials offered by such memory conception, new memory tests have to be created. The existing tools such as the widely used Free and Cued Selective Reminding Test (Buschke, 1984), are not devised to take into consideration the natural dynamic nature of memory. An interesting option lies in the creation of memory tests with items generated by patients themselves based on their personal experiences (Noel et al., 2014).

The aim of a neuropsychological assessment is not only describing the cognitive and behavioral effects of brain damage (for example, identification of memory difficulties after hippocampus impairment). It also serves at understanding the functional implications of a given brain damage (forgetting to take medications, the name of grandchildren…) and to provide possible remediation (such as spaced retrieval training). In our point of view, adopting a more dynamic conception of memory is also of interest with regard to rehabilitation. As a concrete example, let’s consider the case of a patient presenting memory difficulties due to brain injuries. Unlike multisystem models, dynamic and integrative conceptions posit the existence of an integrated memory structure that can be accessed by different modalities. It could thus be interesting to propose metacognitive training or other techniques that could help the patient to find the most efficient modality for entering or engaging the integrated memory structure. Moreover, by considering memory as an adaptive function that can be updated in the light of new experiences or changes, we believe that an efficient work on deficit awareness could be performed. Indeed, like in the process of change in various psychotherapies, the clinical neuropsychologist could bring the patient to integrate information on his/her actual state through the process of reactivation of old memories to include new information via the process of reconsolidation (Lane et al., 2015).

The current trend in clinical neuropsychology is to favor a more collaborative interaction between the clinical neuropsychologist and the patient in order to elucidate aspects of the patient’s psychological life not captured by standardize methods. As posited above, a dynamic, functional and integrative conception could be well fitted within this emerging collaborative approach.

## Conclusion

The representation of memory in the brain remains an unresolved issue with long lasting fundamental disputes among researchers. Debating on theories is of major importance and is a bulwark against dogmatism. In the field of research, knowledge on memory is highly dynamic and keeps on evolving. The current means at our disposal to investigate memory (such as neuroimaging technics) have also substantially challenged and modified the traditional structural conception of memory. More integrative and dynamic conceptions of memory have emerged, revitalizing the debate on the representation of memory in the brain. With regard to clinical neuropsychology, currently the structural approach of memory remains dominant. Although this approach has proved its efficacy particularly in diagnosis of illness (like Alzheimer disease), it has nonetheless various limits as outlined previously. The conception of memory as a changing organization in the form of traces, which reflects the brain’s dynamic reality in the tangle of other cognitive functions is well ascribed in research field. Although, such conception has not yet gained its milestones in the dynamic context of clinical investigation of memory, in the present perspective article, we put forward its potential clinical utility. We therefore encourage researchers and clinicians to test in the field of clinical neuropsychology, the usefulness and explanatory power of more dynamic and functional representation of memory in the brain in order to demonstrate more objectively its validity outside the research loop.

## Author Contributions

YG: writing of the article and elaboration of ideas. MH: Elaboration of ideas and verification of the whole manuscript. FC: Elaboration of ideas and verification of the whole manuscript. VQ: Coordinator of the manuscript and elaboration of ideas.

## Conflict of Interest Statement

The authors declare that the research was conducted in the absence of any commercial or financial relationships that could be construed as a potential conflict of interest.
